# Evaluation of the Impact and Implementation of Social Prescribing in Primary Healthcare Units in Lisbon: A Mixed-Methods Study Protocol

**DOI:** 10.5334/ijic.5592

**Published:** 2021-06-21

**Authors:** Louíse Viecili Hoffmeister, Mariana Fortuna Nunes, Cristiano Emanuel Marta Figueiredo, Andreia Coelho, Mariana Filipa Fraga Oliveira, Paula Massano, Ana Gama, Pedro Aguiar, Sónia Dias

**Affiliations:** 1 Public Health Research Centre, National School of Public Health, Universidade NOVA de Lisboa, Avenida Padre Cruz, 1600-560, Lisboa, Portugal; 2 Comprehensive Health Research Center (CHRC), Campo Mártires da Pátria, 130 1169-056 Lisboa, Portugal; 3 Central Lisbon Health Center Cluster, Rua Carvalho Araújo, 103 1900-181 Lisboa, Portugal

**Keywords:** social prescribing, complex interventions, integrated care, health promotion, study protocol, public health

## Abstract

**Background:**

Social Prescribing (SP) is an intervention to link users of the primary healthcare services to non-clinical organizations based on the community to tackle social determinants of health. Despite the potential benefits of SP, the effectiveness of this complex intervention remains uncertain. This paper presents the study protocol of the evaluation of the first SP project in Portugal.

**Methods:**

A mixed-methods study will be conducted to evaluate the SP project. For the quantitative component, a longitudinal, prospective study with a pre-post design will be performed. Data on patients referred to SP will be collected in four different points in time throughout the intervention, using questionnaires on patients’ health status and sociodemographic characteristics, and scales on patients’ well-being, quality of life and activation. The secondary data will be collected using patients’ medical records and SP’s forms about the referral and social responses elaborated within the intervention. Semi-structured interviews with patients and focus groups with stakeholders will be conducted to assess experiences of participation and improvement suggestions on SP.

**Conclusion:**

Comprehensive and complementary evidence will provide insights and learning for the implementation of future SP interventions. This can contribute to inform policy and practice, and to increase investment in social prescribing interventions.

## Background

The health of individuals is broadly acknowledged to be influenced by a combination of multilevel interrelated determinants – genetic, psychological, lifestyles, ecosystems, as well as social, economic, cultural and structural (including healthcare system) [1]. These multiple complex factors, along with daily life contexts, frequently translate into health inequities [1].

It has been evidenced that situations of loss of employment, poor social and economic resources, social isolation, interpersonal problems, or illness of a relative can trigger the manifestation of physical symptoms, stress, depression, anxiety [2]. Moreover, situations of social exclusion and economic disadvantage are associated with low levels of health literacy, less means for healthy lifestyle habits and decreased access to healthcare, that can lead to non-communicable diseases, and, consequently, to higher mortality and lower life expectancy [3,4,5]. In these cases, an exclusively clinical approach may not be the most appropriate response [2,6].

Social Prescribing (SP) has a potential role in effectively tackling social determinants of health and in preventing exacerbation of pre-existing diseases, with the purpose of improving users’ health and well-being [6,7]. SP is a complex intervention and a solution to respond to non-clinical needs of individuals and to articulate healthcare with other sectors of society [6]. This intervention has been considered an innovative public health approach that allows the provision of integrated care by linking healthcare services to the tertiary sector, and thus providing more than solely traditional health care [8,9]. Furthermore, SP can contribute to reduce inequities by covering and addressing the needs of vulnerable populations (such as migrants, individuals with mental illness, or at risk of social isolation) [10,11,12].

The process of SP starts with the identification of a patients with social, emotional or practical needs by a health professional. These patients include people with one or more chronical medical conditions, who have complex social needs which affect their health and well-being, who are lonely or isolated, or who need support related to mental health [10]. The health professional refers the patient to a link worker, who is responsible for, in a SP appointment, assessing the patient’s needs in detail and developing a tailored plan of activities in collaboration with the patient [13]. The link worker is a key professional in the SP process and provides personalised support to individuals, connecting people to community groups and statutory services [10]. Some of the link worker skills are ability to listen, ability to support people in a way that inspires trust and confidence, ability to communicate effectively with all stakeholders, and commitment to collaboratively working with all local agencies [10]. The link worker helps patients and their carers to navigate the voluntary and community services [8].

Based on the identified needs and the co-designed plan, the patient may be referred to a diverse set of social responses from the community resources, such as physical activities, dance groups, arts and crafts, professional training, education activities, food bank support, day-care centre, volunteering, among others. These responses can be provided by different institutions, including local authorities, social services, community and voluntary organizations [10].

SP has been broadly implemented in the English National Health Service (NHS) as an intervention of the NHS Long-Term Plan since 2019 [10,14]. One of the few randomized clinical trials conducted to evaluate SP has showed improvements in anxiety, pain and self-rated overall health, although no significant changes in depression levels. Higher prescription of medications and higher costs were also found in the intervention group [15]. Another study conducted in the United Kingdom evidenced an increase of self-reported physical activity, a better self-perception of quality of life, well-being and lower BMI in patients enrolled in SP activities. Nevertheless, the authors did not found differences in frequency of alcohol consumption and smoking status among these patients [16]. Other studies also seem to indicate that SP has the potential to promote empowerment and activation for health, in terms of increased knowledge, confidence, motivation and ability of the users to self-manage their health [17,18].

The available research does not provide consistent evidence on the impact of SP. Several studies do not present significative differences in anxiety, depression and well-being levels between control groups and groups of patients enrolled in SP [11,19]. Furthermore, a lack of significative differences in SP’s impact between baseline and follow-up evaluation moments has also been evidenced [20]. The main limitations presented in these studies are small sample sizes, no use of inferential statistics and low response rates to the evaluation instruments, which compromises the quality of the evidence provided [11].

Evidence on the provision of integrated care and networking in SP interventions is scarce, although some policy reports suggest that SP may reinforce the proximity between the social and healthcare sectors by having the participation of local organizations in SP as key-partners in the implementation of personalized, local and preventive activity plans for the patients [10]. Other publications report that SP can also increase the patients’ sense of belonging to the community, in addition to fostering a more efficient local economy [6,11].

In terms of the health system, although contradictory results are often found, some studies indicate that SP can have an impact in the reduction of the number of appointments with a General Practitioner (GP) and in decreased prescription of antidepressant, hypnotic, and anxiolytic medicines for patients enrolled in this type of intervention [10,21]. Other research shows a greater workload of professionals involved in SP and higher costs of referral to this intervention [11].

SP evaluation studies are still incipient and present controversial results. When it comes to the evaluation of SP, a variety of studies has been performed, comprising randomized controlled trials, case-control studies, case studies, qualitative studies, among others, that used various data collection instruments applied with different periodicities, and measured different outcomes [19,20,22,23,24,25]. Indeed, although the wider health benefits of the SP intervention, such as improve patients’ health, well-being and quality of life, should be covered, other outcome measures can be added according to the specific objectives of each intervention [10,20]. Thus, this heterogeneity poses a great challenge when designing SP evaluation studies [20,26,27]. In addition, since SP is a complex intervention, evaluation studies that give insights on its impact for the patients, for the health system and for the community would be valuable [10].

### SP in Portugal

The first SP project in Portugal was launched in two Primary Healthcare Units – *Baixa and Almirante Family Health Units –* located in central Lisbon and provide healthcare to approximately 27500 patients with a high variability of sociodemographic characteristics. All users registered in these Primary Healthcare Units can be referenced to SP, except patients with severe and uncontrolled mental health issue.

The intervention starts when a healthcare professional (general practitioner, nurse or psychologist), during an appointment with a patient, identifies social needs, namely related to social isolation, migrant integration, mental health, physical activity, employment and training, or housing issues. After that, the healthcare professional makes an internal referral through an SP online platform to the link worker, which in the Portuguese context is the unit’s social worker. In this platform the healthcare professional fills in the reasons for referral, presence of chronic diseases and brief history of the patient. Afterwards, the patient is encouraged to schedule a SP appointment with the social worker to continue the support of SP.

In the SP appointment, the social worker performs a needs assessment and helps patients to identify the issues that impact on their health and well-being. Then, the social worker develops a tailored intervention plan in collaboration with the patient, based on the person’s priorities, interests, values and motivations [10]. This plan foresees an external response provided by community partners from the health unit’s geographical area or according to the patient’s preference.

In the next stage of the SP pathway, the social worker refers the patient to the key community partners. Communication between the social worker and community partners is made by e-mail containing information about the patient’s needs and the social partner’s availability of response, and through online forms to monitor the activities in which the patient is participating and the frequency of adherence.

Throughout SP intervention, the social worker communicates with the patient through face-to-face SP appointments or phone calls to follow the compliance and satisfaction with the activity plan, and communicates with the healthcare professional to give feedback on the patient´s case. The number of appointments with the link worker depends on each user’s needs, but as documented elsewhere, the SP intervention takes around 4–6 appointments per patient [16,28], although, if necessary, more follow-ups can be scheduled. The average length of the appointments is 30 minutes.

Overall, the SP intervention changed the conventional role of the social worker in Health Units in Portugal. Health professionals usually referred patients to the social work service when the issues were mainly associated with administrative issues in access to social and health benefits, such as support to buy medicines, functional dependency or end of life support. SP intervention allowed to expand the role of the social worker within primary care to include: advice and support on lifestyle changes (physical activities, food habits), community engagement, employment and training, mental health promotion, personalised emotional and social support, as well as connect people to community groups and statutory services.

## Research aims

The aim of this protocol is to evaluate the SP intervention implemented in Primary Healthcare Units of Lisbon, with a focus on patients’, health-social sector collaboration, and health system’ levels.

The specific objectives are to: I) Explore the extent to which the SP intervention leads to enhance patients’ quality of life, well-being, and activation; II) Explore experiences of patients during their participation on SP activities, including positive and negative aspects, and suggestions for improvement; III) Understand the implementation of SP from the perspectives of health professionals, social workers, and community partners; IV) Describe the networking experience of the key actors involved in SP.

## Methods

### Study design

**Figure 1:**
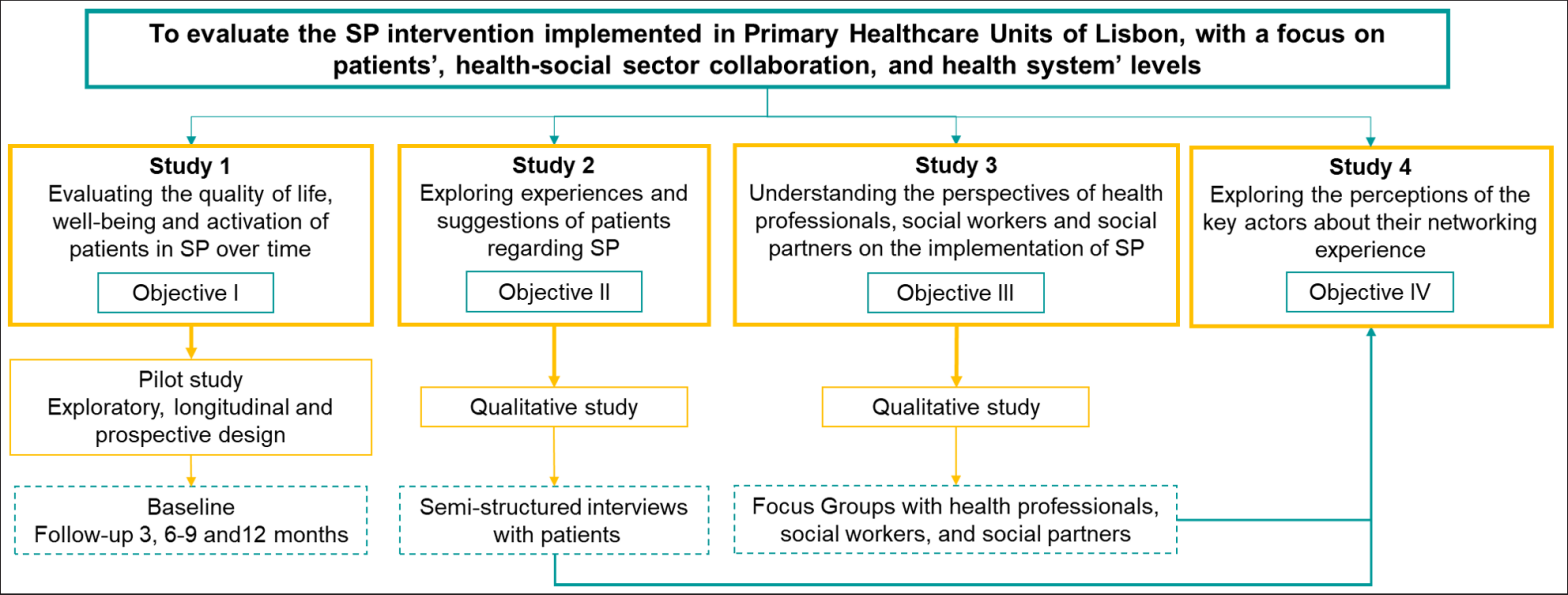
Description of the studies and objectives.

This evaluation protocol will use a mixed-methods approach consisting of four studies, each one addressing a specific objective, as illustrated in ***Figure 1***.

#### Study 1 – Evaluating the quality of life, well-being and activation of patients in SP over time

##### Design and participants

**Figure 2:**
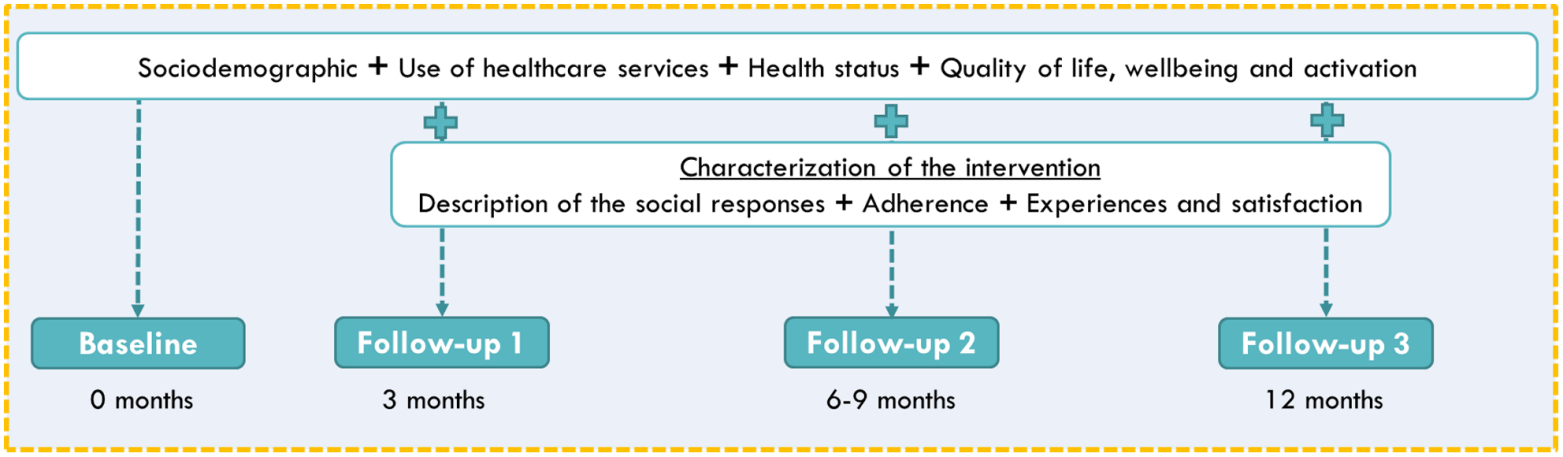
Moments of assessment.

In order to explore the extent to which the SP intervention leads to enhance patients’ quality of life, well-being and activation, a pilot study will be conducted, with an exploratory, longitudinal and prospective design, comprising four assessment moments: baseline, follow-up 1 (third month), follow-up 2 (sixth month) and follow-up 3 (twelfth month) (***Figure 2***).

The sample will include all the patients referred for the first time to a SP appointment who fulfil the inclusion criteria: being ≥18 years old and living in Lisbon. Patients who are terminally ill, who present severe and uncontrolled mental health issues (e.g. uncontrolled schizophrenia, acute psychosis), who are housebound, and who present uncontrolled use of drugs and/or alcohol are excluded from participation in the study. A recruitment target is estimated at 300 patients, with the chances of non-acceptance of participation and losses to follow-up (attrition rate) being estimated at >20%, based on previous studies [15,19,20,28].

All patients referred to SP in the first 12 months will be included in the baseline and the data collection will be conducted during 24 months. Both the patients who undertake SP activities and those who end their participation during data collection period will be included in the follow-ups.

##### Data collection

Before the first SP appointment, the patient will be invited to participate in the study (baseline) and will be asked to provide a written informed consent. ***Table 1*** summarizes the instruments and measures used for data collection throughout the assessment moments.

**Table 1:** Summary table of the evaluation’s quantitative component.


EVALUATION MOMENT	INFORMATION SOURCES	INSTRUMENTS	GROUPS OF VARIABLES	VARIABLES

***Baseline, follow-up* 1, 2 and 3**	Patient	Questionnaire	Sociodemographic characteristics	– Sex – Age – Country of birth – Educational level – Employment status – Number of people in the household – Average monthly income of the household – Preferred Language

			Health status	– Weight and height – Physical activity – Smoking status – Alcohol consumption

			Well-being	– WEMWBS

			Patient’s activation	– PAM13

			Quality of life	– EQ-5D-3L

	Computer system	Health units’ Databases	Health status	– Number of chronic diseases – Types of chronic diseases – Medications consumption

			Use of healthcare services	– Number of appointments in the Primary Healthcare Services – Number of hospital admissions – Number of emergency episodes

***Follow-up* 1, 2 and 3**	Health professionals, social workers and community partners	Forms	Characterization of the intervention	– Type of health professional that made the referral – Motive for referral – Activities provided – Accepted and planned activities – Adherence to the activities


A questionnaire will be applied to collect data on: I) Sociodemographic characteristics (sex, age, country of birth, educational level, employment status, number of people in the household, average monthly income of the household and preferred language); II) Health status (height and weight, physical activity, smoking behaviour, and alcohol consumption); III) Mental well-being (using the Warwick-Edinburgh Mental Well-being Scale – WEMWBS); IV) Patient activation (through 13-item patient activation measure – PAM13); and V) Quality of life (using the EuroQol 5 dimensions instrument – EQ-5D-3L). Prior authorization for the use of each of the scales in Portuguese and English versions was obtained.

The questionnaire was tested in a purposive sample of patients not enrolled in the study. As a result, the guidelines for filling the questionnaire were simplified in order to ensure clarity of the information.

In addition to the questionnaire, secondary data will be collected from patients’ medical records. This data will consist of indicators related to the use of healthcare services (number of appointments in the Primary Healthcare Units, of hospital admissions, and of emergency episodes) and health status (number and types of chronic diseases, and medications consumption).

In the follow-ups, data will be collected using the same instruments of baseline. The questionnaire will be applied in the health unit before the SP appointments. In order to ensure the inclusion of people with low literacy and educational levels or that experience difficulties with self-administration, the questionnaire will be administered by a researcher through an interview (either in Portuguese or in English). In situations where the patient has interest in participating in the study but is unable to communicate in Portuguese or English, or has learning disabilities and other types of disabilities, a person that accompanies the patient can help in collecting the information, with the consent of the patient and when applicable. A unique alphanumeric code will be used to identify each participant in all evaluation instruments throughout the longitudinal assessment moments.

Additional information about the SP intervention will comprise data on the type of health professional that made the referral and motive for signposting (from the SP form filled in by the health professional) and information about the SP activities provided, accepted and programmed, and patient’s adherence to the activities (through the online forms filled in by the community partners).

##### Data analysis

The primary outcome measures comprise the levels of quality of life, well-being and the patients’ activation. The sociodemographic characteristics, health status, use of healthcare services and characteristics of the intervention are considered the secondary outcome measures. A statistical analysis will be conducted to analyse the association between primary and secondary outcomes. Furthermore, comparative measures between the baseline and follow-up 1, 2 and 3 will be computed, using generalized estimating equations (GEE) [29]. All analyses will be conducted using the IBM SPSS Statistics software, in the most recent version available.

#### Study 2 – Exploring experiences and suggestions of patients regarding SP

##### Design and participants

With the purpose of exploring experiences of patients during their participation on SP activities, a qualitative study will be conducted through semi-structured interviews to patients included in study 1.

Participants will be recruited during the follow-up 1 or in SP appointments. In cases where the patients completed the intervention plan or had given up the intervention or follow-up, they will be recruited with the cooperation of the social worker in contacting and inviting them for the study. Selecting patients with different socioeconomic characteristics (such as sex, age, employment status and migration background) and level of attendance to the intervention will help to obtain a diverse sample and distinct perspectives on this intervention.

Both the patients that are still in SP and those who had concluded their participation (i.e. who completed the SP intervention plan or who gave up the intervention) will be included. Those with significant hearing impairments, or with insufficient Portuguese/English speaking skills (when unaccompanied by a translator) will be excluded from participation.

##### Data collection

The participants will be contacted via phone call or email and invited to participate in a face-to-face interview. Interviews will take place in primary healthcare facilities or other location according to participants’ preference and availability.

A semi-structured guide will be used to conduct the interview with patients and will include questions about characteristics of the SP intervention, experiences on the SP activities performed, perceived changes in lifestyle, perspectives about positive and negative aspects of SP, opinions on the interactions with healthcare professionals and community partners, patient’s satisfaction with the SP intervention and suggestions for improvement. All interviews will be audio recorded.

Participants will be recruited until no new themes emerge from the data collected (data saturation) [30]. We estimate to interview 10 to 15 patients in each of the Primary Healthcare Units involved in the study.

##### Data analysis

Audio records of the interviews will be transcribed verbatim. Participants will revise the transcripts of their interviews for validation, to ensure its accuracy. Then, the transcripts will be anonymized for storage and scientific publication purposes. The transcripts of the interviews will be analysed through content analysis technique, as described by Bardin [31].

#### Study 3 – Understanding the perspectives of health professionals, social workers and community partners on the implementation of SP

##### Design and participants

Aiming to further understand the implementation of SP a qualitative study will be conducted through focus groups with health professionals (namely GPs, nurses and psychologists), social workers, and community partners involved in SP. The focus group technique, through group interaction, allows to broaden the understanding of the subject under study, its complexity and dynamic character, providing rich, valuable information and collective perspectives, and has been increasingly used in SP evaluation studies [32].

##### Data collection

In order to get the perspective of the stakeholders who have a role in the PS process, and based on the number of professionals involved in the SP intervention in Lisbon, five focus groups will be conducted: two focus groups with health professionals (namely GPs, nurses and psychologists), one focus group with social workers, and two focus groups with community partners. Each focus group will include 6–8 participants, resulting in an approximate total of 30 participants. Focus groups will be conducted by a moderator and a co-moderator of the research team.

A semi-structured guide will be used, covering common topics for all groups regarding perspectives on the process of implementing SP, barriers and facilitators, suggestions for improvement and experiences of collaboration and networking. In addition, each group will discuss about topics specifically related to their role in the SP intervention. The focus group sessions will be audio recorded.

##### Data analysis

Audio records of the focus groups will be transcribed verbatim. Participants will revise the transcripts of their focus group discussion for validation, to ensure the accuracy of the transcription. The transcripts will be anonymized for storage and scientific publication purposes. Content analysis will be performed to analyse the data, as described by Bardin [31].

#### Study 4 – Exploring the perceptions of the key actors about their networking experience

##### Participants, data collection and analysis

This study aims to explore the perceptions of the key actors involved in SP about their interactions and experience of collaboration within the intervention. This study draws on data collected in study 2 (interviews with patients) and study 3 (focus groups with health professionals, social workers, and community partners).

This study will focus on the collaboration between key actors, namely the interactions and exchange of information, the development of new responses, the challenges, lessons learned and perceived added value of acting in an intersectoral network.

The procedures for data treatment and analysis of the interviews and the focus group discussions are described in Study 2 and 3, respectively.

### Ethical considerations

Ethical approval for this evaluation study was obtained from the Ethics Committee of the Regional Health Administration of Lisbon and Tagus Valley (nº 5 2020/CES/2020).

In all studies, potential participants will be informed about the study’s objectives, data collection procedures, benefits and potential risks of participation. Participants will be assured that their participation in the study is voluntary and anonymous, and that the collected data is confidential. Participants will also be assured that refusing to participate or withdrawing consent at any moment will not entail any consequences, nor interfere with their participation in SP activities. After each participant agrees to participate, he/she will be asked to read and sign the informed consent. In study 1, this will take place prior to the first SP appointment, and in the studies 2 and 3 before the interviews and focus groups, respectively.

In study 1, the unique alphanumeric code will ensure that participation in the study is anonymous, and only the healthcare units’ professional will have the information needed to link the codes with patients’ personal information (name and contacts). The secondary data will be extracted, anonymized, processed and stored by the healthcare units’ professional, and then made available to the research team.

In studies 2, 3 and 4, an authorization to audio records the interviews and focus groups will be requested to each participant. The interviews and focus groups will be conducted in a private room to ensure the comfort of the participants and avoid interruptions.

Electronic data (data bases, computer files) will be stored as protected files with exclusive access of the research team. Paper-based data (questionnaires and consent forms) will be stored in locked cabinets in secure offices, only accessible by the research team. The identifiable information, such as participants’ names, will not be included in the transcriptions.

## Discussion

This evaluation protocol covers the three major dimensions where SP has potential impact: the patients, the collaboration between the health and social sectors, and the health system. This will be accomplished through triangulation of methods and information sources (quantitative measures along with in-depth interviews and focus groups) based on all key actors’ perspectives. Using such a broad evaluation scope will enable to obtain a diverse data corpus, allowing to explore in more depth the complexity of SP and its effects [33]. In fact, due to the interactions that occur between the various components of SP, as well as the number of groups or organisational levels involved, and the variability of outcomes, evaluating this kind of interventions implies to consider the context, the characteristics, the implementation process, the mechanisms of impact and the outcomes of the intervention [34].

In the patient’s dimension, the use of standardized, validated and repeated measures throughout time will provide a quantitative overview of SP’s contribution to individuals’ quality of life, well-being and activation. The WEMWBS, EQ-5D and PAM13 scales are the most frequent measuring tools used in SP evaluation studies [11,20]. Hence, the application of these scales, validated for the Portuguese context, increases the comparability with other studies. Furthermore, the number of assessment moments and long follow-up duration address weaknesses previously pointed out by several authors [11,20,33], since single and short (up to 4 months post intervention) follow-ups have been more commonly applied. A long follow-up interval has been considered essential to consistently measure the benefits of SP for both patients and the health system [17,35].

Moreover, the qualitative study will enable us to obtain in-depth insights on the patients’ experiences of participation in SP activities. Previous research on impact evaluation show that a qualitative approach can reveal strong positive narratives about the impact of SP in patients’ lives (such as improvements in feelings of well-being), even if the quantitative outcomes do not reflect these improvements (such as improvements in feelings of loneliness and social isolation) [19,20].

The SP’s impact on the collaboration between the health and social sectors will also be evaluated. Specifically, SP interventions and social responses provided to patients, and accepted and performed by them will be assessed using information from the quantitative, longitudinal study. The focus groups will provide information on articulation and communication between both sectors. Additionally, it will provide an insight into the health professionals’, social workers’ and community partners’ perspectives, which has only been obtained in few studies and remains an important gap in the literature regarding SP evaluation [19,20].

More broadly, the collection of information about the use of healthcare services will enable an evaluation of the influence of SP at the health system level. The relevance of assessing SP’s effects on this dimension has been previously highlighted [21,24]. SP responds to social needs that often lead patients to seek solutions in healthcare services. Thus, by providing effective responses to these needs, SP can contribute to reduce the overuse of healthcare services and the health professionals’ workload.

Besides the SP’s impact, this evaluation study also intends to provide useful knowledge on its process of implementation. The follow-up of all patients referred to SP, including those who conclude or withdraw their participation from SP activities before the follow-up evaluations represents an important innovation of this study. This will shed light on barriers to adherence to SP’s activities, while also allowing a comparison of outcomes between adherent and non-adherent patients.

Another differential of this evaluation study is that information on activities provided by community partners, accepted by patients and performed within SP will be collected and monitored for each participant. This will allow to collect detailed information about each patient’s pathway within SP, which has been described as a limitation in several studies [11,17]. It is acknowledged that the use of this approach is crucial to understand both the scope and the acceptability of the intervention among patients [34].

Moreover, this evaluation study also covers the experience of collaboration and perceived added value of intersectoral networks composed of different key actors. These topics have not been broadly addressed in published studies, and this exchange of information between the different levels of service provision – social and health sectors – can be valuable for the success of the SP intervention [33,36,37,38].

An expected limitation of this study is the challenges in generalizing the obtained results, since this is an evaluation of an SP intervention implemented in a local and specific context. The lack of a control group is a limitation of the Study 1 and therefore the results of this study will need to be interpreted with caution. It will be beneficial to conduct controlled studies in the future. Another limitation, previously described by other authors, is the possible loss of participants in the follow-up assessments [19,20], mainly in the last one (12 months) [28,39]. This possibility can arise from difficulties in contacting patients or non-attendance to scheduled appointments. Nevertheless, the findings of this study can contribute to assist the planning and implementation of SP and its evaluation in other contexts and countries.

## Conclusions

This study protocol is designed to conduct a robust assessment of the SP intervention by addressing the limitations pointed out in other studies and by comprising the perceptions of the different stakeholders involved in the intervention. Although many studies have assessed the impact of this intervention in the last years, the heterogeneity of study designs and the discrepancy of results make it essential to conduct a tailored and comprehensive evaluation of the intervention.

The results of this study will provide knowledge on the potential impact of the SP intervention for patients, for the collaboration between the health and social sector, and for the health system in the Portuguese context. These results can also contribute to inform practice and policy in national and international contexts, and to provide insights and learning for the implementation of future SP interventions.
